# Privacy-Preserving Integration of Medical Data: A Practical Multiparty Private Set Intersection

**DOI:** 10.1007/s10916-016-0657-4

**Published:** 2017-01-16

**Authors:** Atsuko Miyaji, Kazuhisa Nakasho, Shohei Nishida

**Affiliations:** 1 Graduate School of Engineering, Osaka University, 2-1 Yamadaoka, Suita, Osaka, Japan; 2 Department of Machine Intelligence and Systems Engineering, Akita Prefectural University, 84-4 Ebinokuchi, Tsuchiya, Yurihonjo, Akita Japan; 3 Japan Advanced Institute of Science and Technology, Asahidai 1-1, Nomi-shi, Ishikawa Japan

**Keywords:** Medical data, Privacy-preserving data integration, Private set intersection

## Abstract

Medical data are often maintained by different organizations. However, detailed analyses sometimes require these datasets to be integrated without violating patient or commercial privacy. Multiparty Private Set Intersection (MPSI), which is an important privacy-preserving protocol, computes an intersection of multiple private datasets. This approach ensures that only designated parties can identify the intersection. In this paper, we propose a practical MPSI that satisfies the following requirements: The size of the datasets maintained by the different parties is independent of the others, and the computational complexity of the dataset held by each party is independent of the number of parties. Our MPSI is based on the use of an outsourcing provider, who has no knowledge of the data inputs or outputs. This reduces the computational complexity. The performance of the proposed MPSI is evaluated by implementing a prototype on a virtual private network to enable parallel computation in multiple threads. Our protocol is confirmed to be more efficient than comparable existing approaches.

## Introduction

Medical organizations often store the data accumulated through medical analyses. However, detailed data analysis sometimes requires separate datasets to be integrated without violating patient or commercial privacy. Consider the scenario in which the occurrence of similar accidents can be attributed to a particular defective product. Such defective products should be identified as quickly as possible. However, the databases related to accidents are maintained separately by different organizations. Thus, investigating the causes of accidents is often time-consuming. For example, suppose child *A* has broken her/his leg at school, but it is not clear whether the accident was caused by defective equipment. In this case, information relating to *A*’s injury, such as the patient’s name and type of injury, are stored in hospital database *S*_1_. Information pertaining to *A*’s accident, such as their name and the location of the swing at the school, are stored in database *S*_2_, which is held by the fire department. Finally, information relating to the insurance claim following *A*’s accident, such as the name and medical costs, is maintained in the insurance company’s database, *S*_3_. Computing the intersection of these databases, *S*_1_∩*S*_2_∩*S*_3_, without compromising privacy would enable us to combine the separate sets of information, which may allow the cause of the accident to be identified. Let us consider another situation. Several clinics, denoted as P_*i*_, maintain separate databases, represented as *S*_*i*_. The clinics wish to know the patients they have in common to enable them to share treatment details; however, P_*i*_ should not be able to access any information about patients not stored in their own dataset. In this case, the intersection of the set must not reveal private information.

These examples illustrate the need for the Multiparty Private Set Intersection (MPSI) protocol [11, 17, 18, 21]. MPSI is executed by multiple parties who jointly compute the intersection of their private datasets. Ultimately, only designated parties can access the intersection. Previous protocols are impractical, because the bulk of the computation is a function of the number of players. One previous study required the size of the datasets maintained by the different players to be equal [17, 21]. Another study [11] computed only the approximate number of intersections, whereas other researchers [18] required more than two trusted third-parties.

In this paper, we propose a practical MPSI with the following features:

1. The size of the datasets maintained by each party is independent of those maintained by the other parties.
2. The computational complexity for each party is independent of the number of parties. This is accomplished by introducing an outsourcing provider, $\phantom {\dot {i}\!}\mathcal {O}$. In fact, all computations related to the number of parties are carried out by $\phantom {\dot {i}\!}\mathcal {O}$. Thus, the number of parties is irrelevant.

The remainder of this paper is organized as follows. Previous results that are used to develop the proposed protocol are summarized in “Preliminaries”. “Previous work” then introduces some related studies. We propose the new MPSI in “Practical MPSI”, and present the results of its implementation in “Implementation results”.

## Preliminaries

In this section, we summarize the DDH assumption, Bloom filter, and ElGamal encryption. We consider security according to the honest-but-curious model [13]: all players act according to their prescribed actions in the protocol. A protocol that is secure in an honest-but-curious model does not allow any player to gain information about other players’ private input sets, besides that which can be deduced from the result of the protocol. Note that the term *adversary* here refers to insiders, i.e., protocol participants. Outsider adversaries are not considered. In fact, behavior by outsider adversaries can be mitigated via standard network security techniques.

Our protocol is based on the following security assumption.

### **Definition 1**

(**DDH Assumption**) Let *t* be a security parameter. A decisional Diffie–Hellman (DDH) parameter generator $\phantom {\dot {i}\!}\mathcal {I}\mathcal {G}$ is a probabilistic polynomial time (ppt) algorithm that takes input 1^*k*^ and outputs a description of a finite field $\phantom {\dot {i}\!}\mathbb {F}_{p}$ and a basepoint $\phantom {\dot {i}\!}g \in \mathbb {F}_{p}$ with prime order *q*. We say that $\phantom {\dot {i}\!}\mathcal {I}\mathcal {G}$ satisfies the *DDH assumption* if |*p*_1_−*p*_2_| is negligible (in *K*) for all ppt algorithms *A*, where $\phantom {\dot {i}\!}p_{1}= \text {Pr} [ (\mathbb {F}_{p}, g) \leftarrow \mathcal {I}\mathcal {G}(1^{K}); y_{1}=g^{x_{1}}, y_{2}= g^{x_{2}} \leftarrow \mathbb {F}_{p}: A(\mathbb {F}_{p}, g, y_{1}, y_{2}, g^{x_{1}x_{2}}) = 0]$ and $p_{2}={\text {Pr}} [ (\mathbb {F}_{p}, g) \leftarrow \mathcal {I}\mathcal {G}(1^{K}); y_{1}=g^{x_{1}}, y_{2}= g^{x_{2}}, z \leftarrow \mathbb {F}_{p}: A(\mathbb {F}_{p}, g, y_{1}, y_{2}, z) = 0]$.

A Bloom filter [3], denoted by *B* *F*, consists of *m* arrays and has a space-efficient probabilistic data structure. The *B* *F* can check whether an element *x* is included in a set *S* by encoding *S* with at most *w* elements. The encoded Bloom filter of *S* is denoted by *B* *F*(*S*).

The *B* *F* uses a set of *k* independent uniform hash functions $\phantom {\dot {i}\!}\mathcal {H} = \left \{ H_{0}, ..., H_{k-1} \right \}$, where *H*_*i*_:{0,1}^∗^→{0,1,⋯ ,*m*−1} for 0≤∀*i*≤*k*−1. The *B* *F* consists of two functions: *C* *o* *n* *s* *t* embeds a given set *S* into *B* *F*(*S*), and *E* *l* *e* *m* *e* *n* *t* *C* *h* *e* *c* *k* checks whether an element *x* is included in *S*. *S* *e* *t* *C* *h* *e* *c* *k*, an extension of *E* *l* *e* *m* *e* *n* *t* *C* *h* *e* *c* *k*, checks whether an element *x* in *S* ^*′*^ is in *S* ^*′*^∩*S* (see Algorithm 3). In *C* *o* *n* *s* *t* (see Algorithm 1), *B* *F*(*S*) is constructed for a given set *S* by first setting all bits in the array to 0. To embed an element *x*∈*S* into the filter, the element is hashed using *k* hash functions to obtain *k* index numbers, and the bits at these indexes are set to 1, i.e., set *B* *F*[*H*_*i*_(*x*)]=1 for 0≤*i*≤*k*−1. In *E* *l* *e* *m* *e* *n* *t* *C* *h* *e* *c* *k* (see Algorithm 2), we check all locations where *x* is hashed; *x* is considered to be not in *S* if any bit at these locations is 0; otherwise, *x* is probably in *S*.

Some false positive matches may occur, i.e., it is possible that all *B* *F*[*H*_*i*_(*y*)] are set to 1, but *y* is not in *S*. The false positive rate FPR is given by $\phantom {\dot {i}\!}\mathsf {FPR} = \left \{1- \left (1-\frac {1}{m} \right )^{kw}\right \}^{k} \approx \left \{1-e^{-kw/m}\right \}^{k}$ [4]. However, false negatives are not possible, and so Bloom filters have a 100 *%* recall rate. 
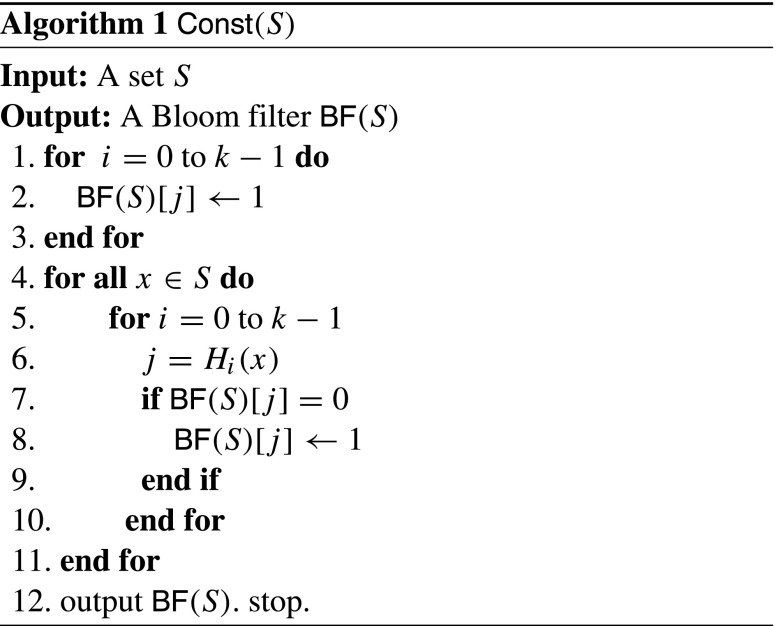
 
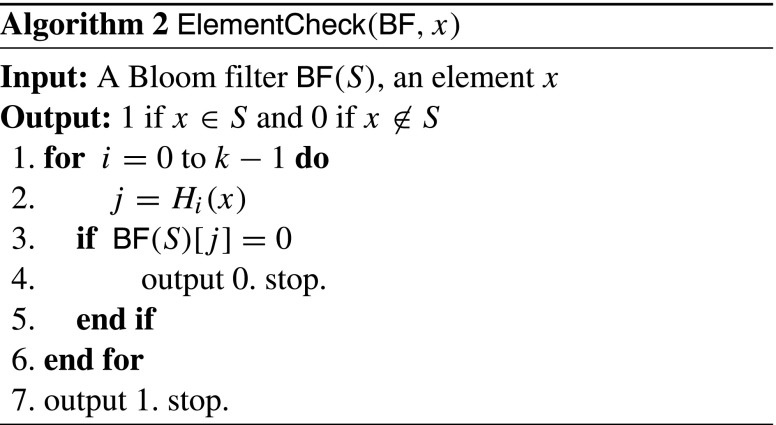
 
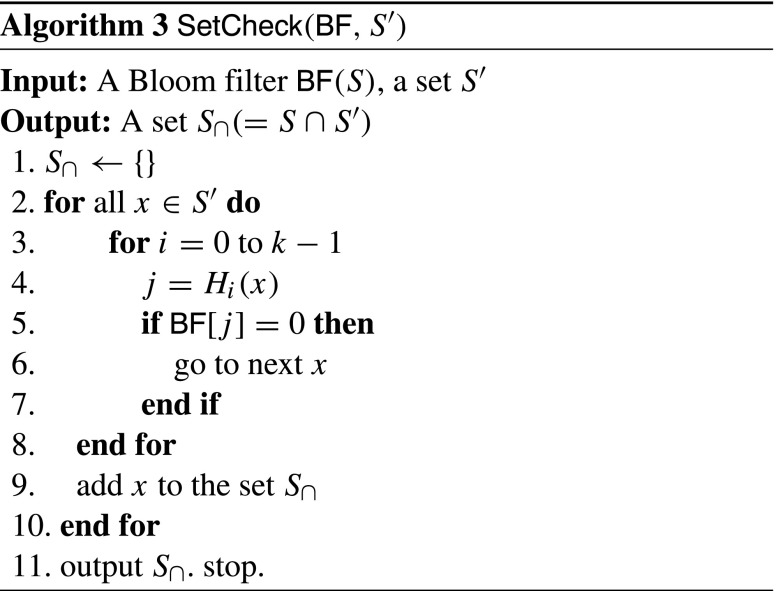


Homomorphic encryption under addition is useful for processing encrypted data. A typical homomorphic encryption under addition was proposed by Paillier [19]. However, because Paillier encryption cannot reduce the order of a composite group, it is computationally expensive compared with the following ElGamal encryption. Our protocol requires matching without revealing the original messages, for which exponential ElGamal encryption (exElGamal) is sufficient [5]. In fact, the decrypted results of exElGamal encryption can distinguish whether two messages *m*_1_ and *m*_2_ are equal, although the exElGamal scheme cannot decrypt messages itself. Furthermore, exElGamal can be used in (*n*,*n*)-threshold distributed decryption [9], where decryption must be performed by *all players acting together*. An exElGamal encryption with (*n*,*n*)-threshold distributed decryption consists of three functions:

### Key generation

Let $\mathbb {F}_{p}$ be a finite field, $\phantom {\dot {i}\!}g \in \mathbb {F}_{p}$, with prime order *q*. Each player P_*i*_ chooses $\phantom {\dot {i}\!}x_{i} \in \mathbb {Z}_{q}$ at random and computes $\phantom {\dot {i}\!}y_{i}=g^{x_{i}} (\text {mod } p)$. Then, $\phantom {\dot {i}\!}y={\prod }_{i=1}^{n}y_{i} (\text {mod } p)$ is a public key and each *x*_*i*_ is a share for each player to decrypt a ciphertext.

### Encryption

*t* *h* *r* *E* *n* *c* [ *m* ]→(*u*,*v*) Choose $r \in \mathbb {Z}_{q}$ at random, and compute both *u*=*g* ^*r*^(mod*p*) and *v*=*g* ^*m*^ *y* ^*r*^(mod*p*) for the input message $\phantom {\dot {i}\!}m \in \mathbb {Z}_{q}$ and a public key *y*. Output (*u*,*v*) as a ciphertext of *m*.

### Decryption

*t* *h* *r* *D* *e* *c*[(*u*,*v*)]→*g* ^*m*^ Each player P_*i*_ computes *z*_*i*_=*u* ^*x*^_*i*_(mod*p*). All players then compute $\phantom {\dot {i}\!}z = {\prod }_{i=1}^{n} z_{i} (\text {mod}{p})$ jointly.^1^ Finally, each player can decrypt the ciphertext as *g* ^*m*^=*v*/*z*(mod*p*).

ExElGamal encryption with (*n*,*n*)-threshold decryption has the following features:

- (1) homomorphic under addition: Enc (*m*_1_)Enc (*m*_2_)=Enc (*m*_1_+*m*_2_) for messages $\phantom {\dot {i}\!}m_{1}, m_{2} \in \mathbb {Z}_{p}$.
- (2) homomorphic under scalar operations: Enc (*m*)^*k*^=Enc (*k* *m*) for a message *m* and $\phantom {\dot {i}\!}k \in \mathbb {Z}_{q}$.

## Previous work

This section summarizes prior works on PSI between a server and a client and MPSI among *n* players. In PSI, let *S*={*s*_1_,...,*s*_*v*_} and *C*={*c*_1_,...,*c*_*w*_} be server and client datasets, where |*S*|=*v* and |*C*|=*w*. In MPSI [17], we assume that each player holds the same number of datasets.

### PSI protocol based on polynomial representation

The main idea is to represent the elements in *C* as the roots of a polynomial. The encrypted polynomial is sent to the server, where it is evaluated on the elements in *S*, as originally proposed by Freedman [12]. This is secure against honest-but-curious adversaries under secure public key encryption. The computational complexity is *O*(*v* *w*) exponentiations, and the communication overhead is *O*(*v*+*w*). The computational complexity can be reduced to *O*(*v*loglog*w*) exponentiations using the balanced allocation technique [1]. Kissner and Song extended this protocol to MPSI [17], which requires *O*(*n* *w* ^2^) exponentiations and *O*(*n* *w*) communication overhead. The MPSI version is secure against honest-but-curious and malicious adversaries (in the random oracle model) using generic zero-knowledge proofs.

### PSI protocol based on DH-key agreement

The main objective here is to apply the DH-key agreement protocol [7]: after representing the server and client datasets as hash values {*h*(*s*_*i*_)} and {*h*(*c*_*i*_)}, respectively, the client encrypts the dataset as $\phantom {\dot {i}\!}\{h(c_{i})^{r_{i}}\}$ using a random number *r*_*i*_ and sends the encrypted set to the server. The server encrypts the client set $\phantom {\dot {i}\!}\{h(c_{i})^{r_{i}}\}$ and the server set {*h*(*s*_*i*_)} using a random number *r*, which gives $\phantom {\dot {i}\!}\{h(c_{i})^{rr_{i}}\}$ and {*h*(*s*_*i*_)^*r*^}, respectively, and returns these sets to the client. Finally, the client evaluates *S*∩*C* by decrypting to {*h*(*c*_*i*_)^*r*^}. This is secure against honest-but-curious adversaries under the DDH assumption. The total computational complexity is *O*(*v*+*w*) exponentiations and the total communication overhead is *O*(*v*+*w*). The security of this approach can be enhanced against malicious adversaries in the random oracle model [6] by using a blind signature. However, no extensions to MPSI based on the DH-key agreement protocol have been proposed.

### PSI protocol based on BF

This protocol was originally proposed in [18]. As the Bloom filter itself reveals information about the other player’s dataset, the set of players is separated into two groups: input players who have datasets and privacy players who perform private computations under shared secret information. In [16], the privacy of each player’s dataset is protected by encrypting each array of the Bloom filter using Goldwasser–Micali encryption [14]. In an honest-but-curious version, the computational complexity is *O*(*k* *w*) hash operations and *O*(*m*) public key operations, and the communication overhead is *O*(*m*), where *m* and *k* are the number of arrays and hash functions, respectively, used in the Bloom filter. The Bloom filter is used in the Oblivious transfer extension [15, 20] and the newly constructed garbled Bloom filter [10]. The main novelty in the garbled Bloom filter is that each array requires *λ* bits, rather than the single bit needed for the conventional Bloom filter. To embed an element *x*∈*S* to a garbled Bloom filter, *x* is split into *k* shares with *λ* bits using XOR-based secret sharing $\phantom {\dot {i}\!}(x=x_{1} \bigoplus ... \bigoplus x_{k})$. The *x*_*i*_ are then mapped to an index of *H*_*i*_(*x*). An element *y* is queried by subjecting all bit strings at *H*_*i*_(*y*) to an XOR operation. If the result is *y*, then *y* is in *S*; otherwise, *y* is not in *S*. The client uses a Bloom filter *B* *F*(*C*) and the server uses a garbled Bloom filter *G* *B* *F*(*S*). If *x* is in *C*∩*S*, then for every position *i* it hashes to, *B* *F*(*C*)[*i*] must be 1 and *G* *B* *F*(*S*)[*i*] must be *x*_*i*_. Thus, the client can compute *C*∩*S*. The computational complexity of this method is *O*(*k* *w*) hash operations and *O*(*m*) public key operations, and the communication overhead is *O*(*m*). The number of public key operations can be changed to *O*(*λ*) using the Oblivious transfer extension. This is secure against honest-but-curious adversaries if the Oblivious transfer protocol is secure. Finally, some researchers have computed the approximate number of multiparty set unions [11].

## Practical MPSI

This section presents a practical MPSI that is secure under the honest-but-curious model.

### Notation and privacy definition

In the remainder of this paper, the following notation is used.

- P _*i*_: *i*-th player, *i*=1,⋯ ,*n*
- $\phantom {\dot {i}\!}\mathcal {O}$: outsourcing provider with no knowledge of the inputs or outputs
- $\phantom {\dot {i}\!}S_{i} = \{ s_{i,1}, s_{i, 2},\cdots , s_{i, w_{i}} \}$: dataset held by P _*i*_, where |*S*_*i*_|=*ω*_*i*_
- ∩*S*_*j*_: intersection of all *n* players
- *t* *h* *r* *E* *n* *c* and *t* *h* *r* *D* *e* *c*: (*n*,*n*)-threshold exElGamal encryption and decryption, respectively
- *m* and *k*: number of arrays and hashes used in *B* *F*
- ***ℓ***=[*ℓ*,⋯ ,*ℓ*] ( 1≤*ℓ*≤*n*): an *m*-dimensional array, where all strings in the array are set to *ℓ*
- *B* *F*(*S*_*i*_)=[*B* *F*_*i*_[0],⋯ ,*B* *F*_*i*_[*m*−1]]: Bloom filter applied to a set *S*_*i*_
- $\mathsf {IBF}(\cup S_{i})=[ {\sum }_{i=1}^{n} \mathsf {BF}_{i}[0], \cdots , {\sum }_{i=1}^{n} \mathsf {BF}_{i}[m-1]]$ : integrated Bloom filter of *n* sets {*S*_*i*_}, where ${\sum }_{i=1}^{n} \mathsf {BF}_{i}[j]$ is the sum of all players’ arrays.

We introduce an outsourcing provider $\phantom {\dot {i}\!}\mathcal {O}$ to reduce the computational burden on all players. The dealer has no information about the elements of any player’s set. The privacy issues faced by MPSI with an outsourcing provider can be informally written as follows.

#### **Definition 2**

(MPSI privacy) An MPSI scheme with an outsourcing provider $\phantom {\dot {i}\!}\mathcal {O}$ is player-private if the following two conditions hold:

- P _*i*_ does not learn anything about the elements of other players’ datasets except for the elements in ∩*S*_*j*_.
- the outsourcing provider $\phantom {\dot {i}\!}\mathcal {O}$ does not learn anything about the elements of any player’s set.

### Proposed MPSI

**Fig. 1:**
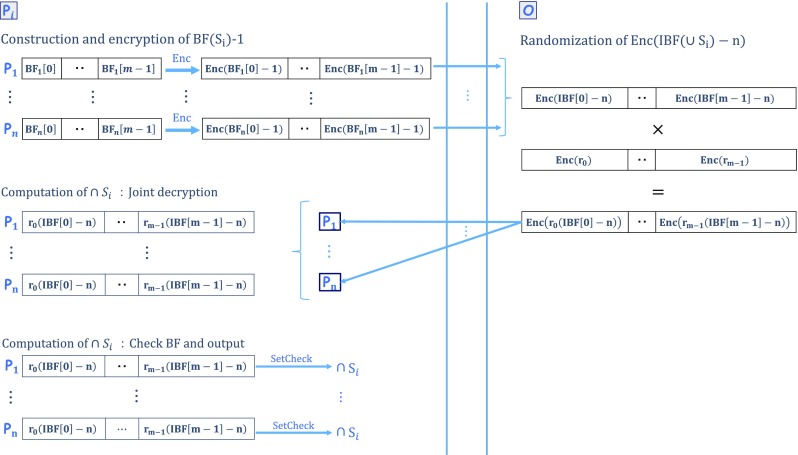
Overview of our MPSI

Our MPSI consists of four phases: i) initialization, ii) Bloom filter construction and the encryption of P _*i*_ data, iii) the $\phantom {\dot {i}\!}\mathcal {O}$’s randomization of *t* *h* *r* *E* *n* *c*(*I* *B* *F*(∪*S*_*i*_)−**n**), and iv) the computation of ∩*P*_*i*_. The computation of ∩*P*_*i*_ consists of three steps: a) joint decryption of an (*n*,*n*)-threshold exElGamal among *n* players, b) Bloom filter check, and c) output intersection. Figure 1 shows an overview of our protocol after the initialization phase. The system parameters of a finite field $\phantom {\dot {i}\!}\mathbb {F}_{p}$ and a basepoint $\phantom {\dot {i}\!}g \in \mathbb {F}_{p}$ with order *q* for an (*n*,*n*)-threshold exElGamal encryption ( *t* *h* *r* *E* *n* *c*, *t* *h* *r* *D* *e* *c*) are provided to both P _*i*_ and $\phantom {\dot {i}\!}\mathcal {O}$. For the Bloom filter, *C* *o* *n* *s* *t*(*S*) and *S* *e* *t* *C* *h* *e* *c* *k*(*B* *F*,*S* ^*′*^) are only provided to P _*i*_, where the array size is *m* and *k* independent hash functions are used.

To encrypt, randomize, or subtract a vector such as a Bloom filter *B* *F*=[*a*_0_,⋯ ,*a*_*m*−1_], each location is encrypted, randomized, or subtracted independently:

$$\begin{array}{@{}rcl@{}} \mathsf{thrEnc}(\mathsf{BF}) &=& [\mathsf{thrEnc}(a_{0}), \cdots, \mathsf{thrEnc}(a_{{m}-1})], \\ \mathbf{r} \mathsf{BF} &=& [r_{0}a_{0}, \cdots, r_{m-1}a_{m-1}], \text{or} \\ \mathsf{BF} - \mathbf{r} &=& [a_{0}-r_{0}, \cdots, a_{m-1}-r_{m-1}] \end{array} $$

for $\phantom {\dot {i}\!}\mathbf {r} = [r_{0}, \cdots , r_{m-1}] \in \mathbb {Z}_{q}^{m}$.

Our protocol proceeds as follows.

**Initialization:**

1. P _*i*_ generates $\phantom {\dot {i}\!}x_{i} \in \mathbb {Z}_{q}$, computes $\phantom {\dot {i}\!}y_{i}=g^{x_{i}} \in \mathbb {Z}_{q}$, and publishes *y*_*i*_ to the other players as a public key, where the corresponding secret key is *x*_*i*_.
2. P _*i*_ computes $\phantom {\dot {i}\!}y={\prod }_{i} y_{i}$, where *y* is an *n*-player public key. Note that no player knows the corresponding secret key $x = \sum x_{i}$ before executing the joint decryption.

**Construction and encryption of** BF(*S*_*i*_) ⋯ **1**:

1. P _*i*_ executes *C* *o* *n* *s* *t*(*S*_*i*_)→*B* *F*(*S*_*i*_) =[*B* *F*_*i*_[0],⋯ ,*B* *F*_*i*_[*m*−1]] (Algorithm 1).
2. P _*i*_ encrypts *B* *F*(*S*_*i*_)−**1** using *t* *h* *r* *E* *n* *c*_*y*_:
  $$\begin{array}{@{}rcl@{}} &&\mathsf{thrEnc}_{\textit{y}}(\mathsf{BF}(S_{\textit{i}}) - \mathbf{1}) \\ &&=\![\mathsf{thrEnc}_{\textit{y}}(\mathsf{BF}_{\textit{i}}[0] \,-\,1), \cdots\!,\ \mathsf{thrEnc}_{\textit{y}}(\mathsf{BF}_{\textit{i}}[\textit{m}\,-\,1]\,-\,1)], \end{array} $$
  where *y* is an *n*-player public key.
3. P _*i*_ sends *t* *h* *r* *E* *n* *c*_*y*_(*B* *F*(*S*_*i*_)−**1**) to $\phantom {\dot {i}\!}\mathcal {O}$.

**Randomization of** thrEnc(IBF(∪*S*_*i*_)−**n**):

1. $\phantom {\dot {i}\!}\mathcal {O}$ encrypts *I* *B* *F*(∪*S*_*i*_)−**n** without knowing *I* *B* *F*(∪*S*_*i*_) using an additive homomorphic feature and multiplying by *t* *h* *r* *E* *n* *c*_*y*_(*B* *F*(*S*_*i*_)−**1**) as follows:
  $$\mathsf{thrEnc}_{\textit{y}}(\mathsf{IBF}(\cup S_{\textit{i}})- \mathbf{n}) = {\prod}_{i=1}^{n} \mathsf{thrEnc}_{\textit{y}}(\mathsf{BF}(S_{\textit{i}})- \mathbf{1}). $$
2. $\phantom {\dot {i}\!}\mathcal {O}$ randomizes *t* *h* *r* *E* *n* *c*_*y*_(*I* *B* *F*(∪*S*_*i*_)−**n**) as $\phantom {\dot {i}\!}\mathbf {r}\ = [r_{0}, \cdots , r_{\textit {m}-1}] \in \mathbb {Z}_{\textit {q}}^{\textit {m}}$:
  $$\mathsf{thrEnc}_{\textit{y}}(\mathbf{r} (\mathsf{IBF}(\cup S_{\textit{i}}) - \mathbf{n})) =(\mathsf{thrEnc}_{\textit{y}}(\mathsf{IBF}(\cup S_{\textit{i}}) - \mathbf{n}))^{\mathbf{r}}. $$
3. $\phantom {\dot {i}\!}\mathcal {O}$ broadcasts *t* *h* *r* *E* *n* *c*_*y*_(**r**(*I* *B* *F*(∪*S*_*i*_)−**n**)) to P _*i*_.

**Computation of** ∩**P**_*i*_:

1. All players decrypt *t* *h* *r* *E* *n* *c*_*y*_(**r**(*I* *B* *F*(∪*S*_*i*_)−**n**)) jointly.
2. P _*i*_ computes *S* *e* *t* *C* *h* *e* *c* *k*(**r**(*I* *B* *F*(∪*S*_*i*_)−**n**),*S*_*i*_) and obtains ∩*S*_*i*_.

The above protocol satisfies the correctness requirement. This is because each array position of *t* *h* *r* *E* *n* *c*_*y*_(**r**(*I* *B* *F*(∪*S*_*i*_)−**n**)) is decrypted to 1, where *x*∈∩*S*_*i*_ is embedded by each hash function; however, each array position for which *x*∉∩*S*_*i*_ is embedded by each hash function is decrypted to a random value.

### Security Proof

The security of our MPSI protocol is as follows.

#### **Theorem 1**

*For any coalition of fewer than n players, MPSI is player-private against an honest-but-curious adversary under the DDH assumption.*

#### *Proof*

The views of P _*i*_ and $\phantom {\dot {i}\!}\mathcal {O}$, that is,

$$\mathsf{thrEnc}_{\textit{y}}(\mathsf{BF}_{\textit{m,k}}(S_{\textit{i}})) \,=\,[\mathsf{thrEnc}_{\textit{y}}(\mathsf{BF}_{\textit{i}}[0]), \cdots\!, \mathsf{thrEnc}_{\textit{y}}(\mathsf{BF}_{\textit{i}}[\textit{m}-1])], $$

are shown to be indistinguishable from a random vector $\phantom {\dot {i}\!}\mathbf {r} = [r_{0}, \cdots , r_{m-1}] \in \mathbb {Z}_{q}^{m}$. Assume that a polynomial-time distinguisher $\phantom {\dot {i}\!}\mathcal {D}$ outputs 0 when the views are presented as a random vector and outputs 1 when they are constructed in MPSI, *t* *h* *r* *E* *n* *c*(*B* *F*_*i*_[0]),⋯ ,*t* *h* *r* *E* *n* *c*(*B* *F*_*i*_[*m*−1]). We show that a simulator $\phantom {\dot {i}\!}\overline {\text {SIM}}$ that solves the DDH assumption can be constructed as follows.

Upon receiving a DDH challenge $\phantom {\dot {i}\!}(\overline {g}, \overline {g}^{\alpha }, \overline {g}^{\beta }, \overline {g}^{\gamma })$, $\phantom {\dot {i}\!}\overline {\text {SIM}}$ executes the following:

1. Set *n*-player public key $\phantom {\dot {i}\!}y = \overline {g}^{\beta }$ and choose random numbers *d*_0_,...,*d*_*m*−1_ and *r*_1_,...,*r*_*m*−1_ from $\phantom {\dot {i}\!}\mathbb {Z}_{q}$.
2. Send
  $$\begin{array}{@{}rcl@{}} [(\overline{g}^{\alpha}, \overline{g}^{d_{0}}_{\cdot} \overline{g}^{r}), (\overline{g}^{\alpha})^{r_{1}},\overline{g}^{d_{1}} \cdot (\overline{g}^{\gamma})^{r_{1}}, {\kern-.5pt}\cdots{\kern-.1pt},{\kern-.5pt} \overline{g}^{d_{m-1}} \cdot (\overline{g}^{\gamma})^{r_{m-1}} ] \end{array} $$
  as $\phantom {\dot {i}\!}\overline {\mathsf {thrEnc}_{\textit {y}}({\mathsf {BF}}_{\textit {m,k}}(S_{i}))}$ to $\phantom {\dot {i}\!}\mathcal {D}$.

If $\phantom {\dot {i}\!}(\overline {g}, \overline {g}^{\alpha }, \overline {g}^{\beta }, \overline {g}^{\gamma })$ is a DH-key-agreement-protocol element, i.e., *γ*=*α* *β*, then $\phantom {\dot {i}\!}\overline {\mathsf {thrEnc}_{\textit {y}}({\mathsf {BF}}_{\textit {m,k}}(\mathrm {S}_{\textit {i}}))}$ is distributed in the same way as when constructed by the MPSI scheme. Thus, $\phantom {\dot {i}\!}\mathcal {D}$ must output 1. If $(\overline {g}, \overline {g}^{\alpha }, \overline {g}^{\beta }, \overline {g}^{\gamma })$ is not a DH tuple, then $\overline {\mathsf {thrEnc}_{\textit {y}}({\mathsf {BF}}_{\textit {m,k}}(S_{\textit {i}}))}$ is randomly distributed, and $\phantom {\dot {i}\!}\mathcal {D}$ has to output 0. As a result, $\phantom {\dot {i}\!}\overline {\text {SIM}}$ can use the output of $\phantom {\dot {i}\!}\mathcal {D}$ to respond to the DDH challenge correctly. Therefore, $\phantom {\dot {i}\!}\mathcal {D}$ can answer correctly with negligible advantage over random guessing. Furthermore, as all inputs of each player are encrypted until the decryption is performed, and decryption cannot be performed by fewer than *n* players, nothing can be learned by any player prior to decryption.

As for the views of *t* *h* *r* *E* *n* *c*_*y*_(**r**(*I* *B* *F*_*m,k*_(∪*S*_*i*_)∖**n**)), the same argument holds. Therefore, for any coalition of fewer than *n* players, MPSI is player-private under the honest-but-curious model. □

### Efficiency

Although many PSI protocols have been proposed, to the best of our knowledge, relatively few have considered the multiparty scenario [11, 17, 18, 21]. Our target is multiparty private set intersection, and the final result must be obtained by *all* players acting together, without a trusted third-party (TTP). Among previous MPSI protocols, the approach in [11] computes only the approximate number of intersections, and that in [18] requires more than two TTPs. In contrast, [21] follows almost the same method as [17] and thus has a similar complexity. The only difference exists in the security model. Hence, we only compare our scheme with that of [17].

The computational and communication efficiency of the proposed protocol and [17] are compared in Table 1. These approaches are secure against honest-but-curious adversaries without a TTP under exElGamal encryption (DDH security) and Paillier encryption (Decisional Composite Residue (DCR) security), respectively.

**Table 1:** Efficiency of [17] and the proposed protocol

	[17]	Ours
Computational complexity	*O*(*n* *ω* ^2^)	*P*_*i*_:*O*(*ω*_*i*_),*O*:*O*(*n* *ω*)
Communication overhead	*O*(*n* *ω*)	*P*_*i*_:*O*(*ω*+*n*),*O*:*O*(*n* *ω*)
Restriction on set size	|*S*_1_|=…=|*S*_*n*_|	none
Protected values	*S*_*i*_(∀*i*∈[1,*n*])	*S*_*i*_,|*S*_*i*_|(∀*i*∈[1,*n*])

Our MPSI uses the Bloom filter for the computations performed by P _*i*_ and the integrations performed by the $\phantom {\dot {i}\!}\mathcal {O}$. The use of a Bloom filter eliminates the restriction on set size. Thus, in our MPSI, the set size of each player is flexible. However, P _*i*_’s computations consist of Bloom filter construction, joint decryption, and Bloom filter check. Neither the computations related to the Bloom filter nor the joint decryption depends on the number of players, as shown in “Preliminaries”. In summary, the computational complexity of operations performed by P _*i*_ is *O*(*ω*_*i*_). All player-dependent data are sent to $\phantom {\dot {i}\!}\mathcal {O}$, who integrates ${\prod }_{i=1}^{n} \mathsf {thrEnc}_{y}(\mathsf {IBF}(\cup S_{i}))$ without decryption. As a result, the computational complexity of operations performed by $\phantom {\dot {i}\!}\mathcal {O}$ is *O*(*n* *ω*).

## Implementation results

### Implementation

To investigate the behavior and performance of our MPSI protocol, we implemented a prototype in C++ using the GNU Multi-Precision (GMP) library (version 5.1.3) and OpenSSL (version 1.0.1f). GMP is used for large-integer arithmetic and random number generation in the exElGamal encryption. To instantiate hash functions for the Bloom filter, we used SHA-1 in OpenSSL: *H*_*i*_(*x*):=sha1(*s*_*i*_ ∥ *x*) mod *m*, where *s*_*i*_ is a unique salt. This truncation of the hash functions is based on the recommendation of the National Institute of Standards and Technology (NIST) [8]. Each executable communicates through TCP. We used Boost.Asio C++ 1.54.0 for the TCP socket.

The C++ prototype has two executables: one for the players and one for the outsourcing provider. The prototype can work in either pipeline or parallel mode. In pipeline mode, the computation and communication threads are separated. Thus, computation and data transmission are processed in parallel when possible. Pipeline mode allows each executable to start immediately without waiting for the completion of all previous computations. Parallel mode extends the pipeline mode by multiplying the number of computation threads in each executable. The most expensive process of our protocol is Bloom filter encryption and decryption. In parallel mode, the encryption and decryption computation is conducted in multiple threads. This significantly improves the performance of our protocol.

### Evaluation

All experiments were performed on the Google Compute Engine (GCE). GCE is a cloud computing system that delivers virtual machines running in Google’s data centers. In our experiments, each executable was calculated on a single virtual machine. We used the Ubuntu 14.04 LTE operating system with Intel Xeon 2.50 GHz CPUs. Each CPU core was assigned 3.75 GB of memory. Every virtual machine was connected to a virtual private network. The bandwidth between two virtual machines was approximately 2.0 Gbps, although our protocol used less than 10 Mbps.

The time required for Bloom filter construction, encryption, decryption, randomization procedures, and MPSI computation was measured. However, the measurements do not include initialization and finalization, e.g., parsing command lines, reading and writing CSV files, TCP socket setup and shutdown, and public key exchange. Each player input a database set of size 2^6^– 2^14^. We measured the performance for *n*=4,8,16 and tested the security parameters for 80-bit, 112-bit, 128-bit, 196-bit, and 256-bit security. Each security parameter is half of the bit size of *q*. The evaluation of the security parameter is based on the NIST guidelines for key management [2], as summarized in Table 2. We chose a false positive rate *F* *P* *R*=0.65 *%*, as was adopted in [18].

**Table 2:** Security parameter and group size

security parameter	|*p*|	|*q*|
80	1024	160
112	2048	224
128	3072	256
192	7680	384
256	15360	512

All numbers shown in the table are in bits

**Fig. 2:**
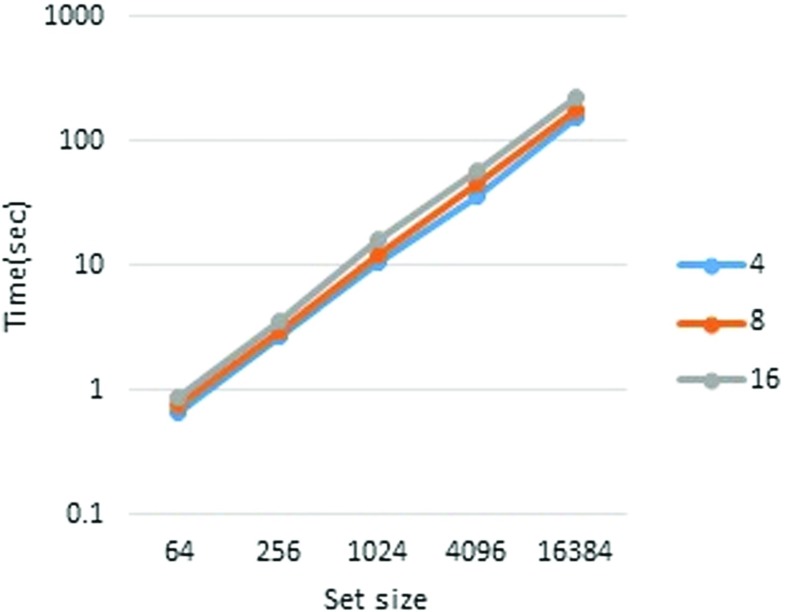
Outsourcing provider, 80-bit security

**Fig. 3:**
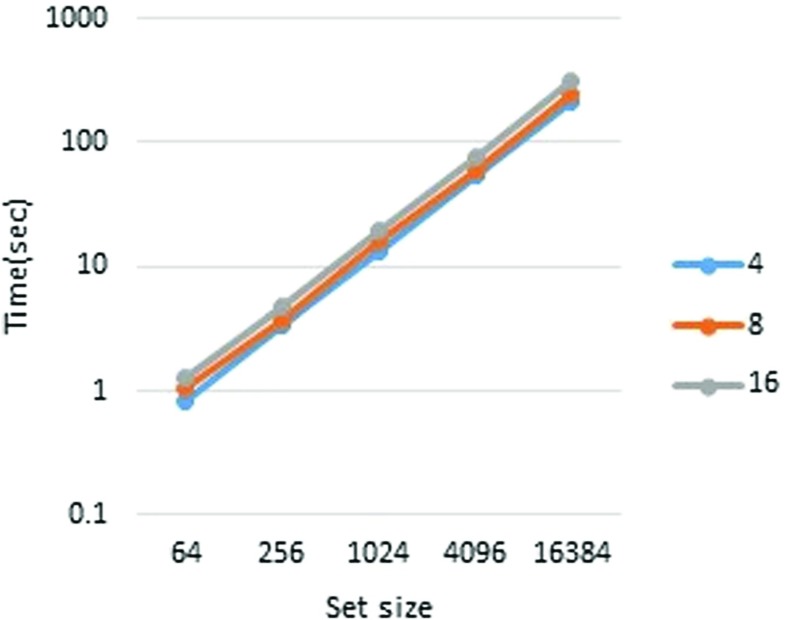
Player, 80-bit security

**Fig. 4:**
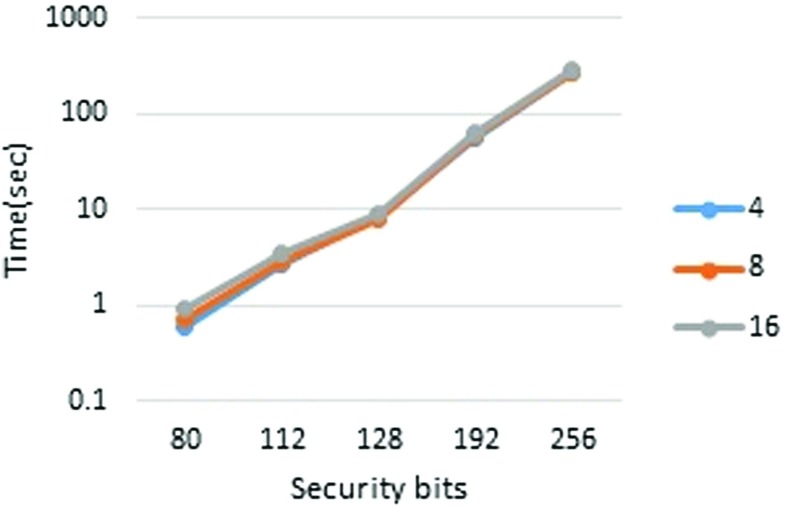
Outsourcing provider, set size = 2^6^

**Fig. 5:**
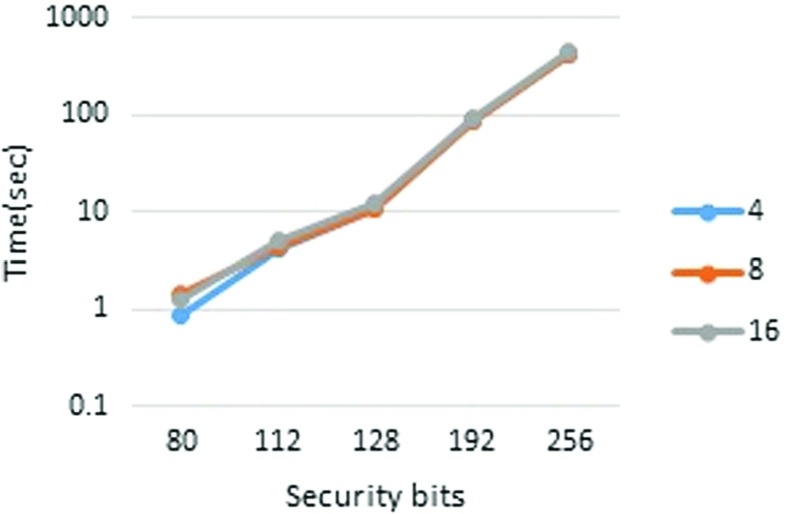
Player, set size = 2^6^

First, we report the runtimes in pipeline mode. The performance measurements are presented in Tables 3 and 4 (Figs. 2, 3, 4, and 5). To measure each executable time separately, we excluded the wait time for communication. From Table 3, it is clear that the runtime scales almost linearly with the set size. It is also apparent that the player’s runtime increases in accordance with *n*. This is because, in our implementation, each player performs the joint decryption process independently. However, the joint decryption process can be distributed by the players so that the computational complexity remains constant with respect to *n*. The outsourcing provider’s runtime obeys scales with the computational complexity, namely, *O*(*n* *ω*). The breakdown of runtimes is presented in Tables 5 and 6.

**Table 3:** Pipeline mode performance (80-bit security)

*n*	exe	Set size				
		2^6^	2^8^	2^10^	2^12^	2^14^
4	$\phantom {\dot {i}\!}\mathcal {O}$	0.65	2.69	10.4	36.7	151
	*P*	0.82	3.39	13.4	54.1	214
8	$\phantom {\dot {i}\!}\mathcal {O}$	0.76	2.95	12.4	44.4	178
	*P*	0.90	3.75	15.7	60.3	241
16	$\phantom {\dot {i}\!}\mathcal {O}$	0.90	3.64	15.8	56.4	225
	*P*	1.30	4.71	19.2	76.1	307

All times in the table are in seconds

**Table 4:** Pipeline mode performance (set size = 2^6^)

*n*	exe	Security parameter (bit)				
		80	112	128	192	256
4	$\phantom {\dot {i}\!}\mathcal {O}$	0.61	2.74	8.29	57.2	275
	*P*	0.87	4.28	11.1	85.7	417
8	$\phantom {\dot {i}\!}\mathcal {O}$	0.72	2.95	7.84	58.1	277
	*P*	1.43	4.38	10.8	86.9	417
16	$\phantom {\dot {i}\!}\mathcal {O}$	0.90	3.41	9.09	61.4	284
	*P*	1.30	5.18	12.0	91.8	433

All times in the table are in seconds

**Table 5:** Breakdown of runtime (set size = 2^6^, *n*=4)

exe	Process	Security parameter (bit)				
		80	112	128	192	256
$\phantom {\dot {i}\!}\mathcal {O}$	(A)	0.61	2.74	8.29	57.2	275
*P*	(B)	0.50	2.67	6.79	55.8	275
	(C)	0.37	1.60	4.35	29.9	142
	(D)	∼ 0.01	∼ 0.01	∼ 0.01	∼ 0.01	∼ 0.01

All times in the table are in seconds

**Table 6:** Breakdown of runtime (set size = 2^6^, Security parameter = 80)

exe	Process	Number of Players		
		4	8	16
$\phantom {\dot {i}\!}\mathcal {O}$	(A)	0.55	0.67	0.82
*P*	(B)	0.45	0.44	0.44
	(C)	0.34	0.43	0.67
	(D)	∼ 0.01	∼ 0.01	∼ 0.01

All times in the table are in seconds

The processes described in the table are as follows:

- Outsourcing provider
- (A) Randomization of *t* *h* *r* *E* *n* *c*(*I* *B* *F*(∪*S*_*i*_)−**n**)
- Player
- (B) Construction and encryption of *B* *F*(*S*_*i*_)−**1**
- (C) Joint decryption of *t* *h* *r* *E* *n* *c*_*y*_(**r**(*I* *B* *F*_*m,k*_(∪*S*_*i*_)−**n**))
- (D) *S* *e* *t* *C* *h* *e* *c* *k*(**r**(*I* *B* *F*(∪*S*_*i*_)−**n**),*S*_*i*_) and obtains ∩*S*_*i*_

Clearly, the time consumption is dominated by the encryption and decryption of the Bloom filter array.

**Fig. 6:**
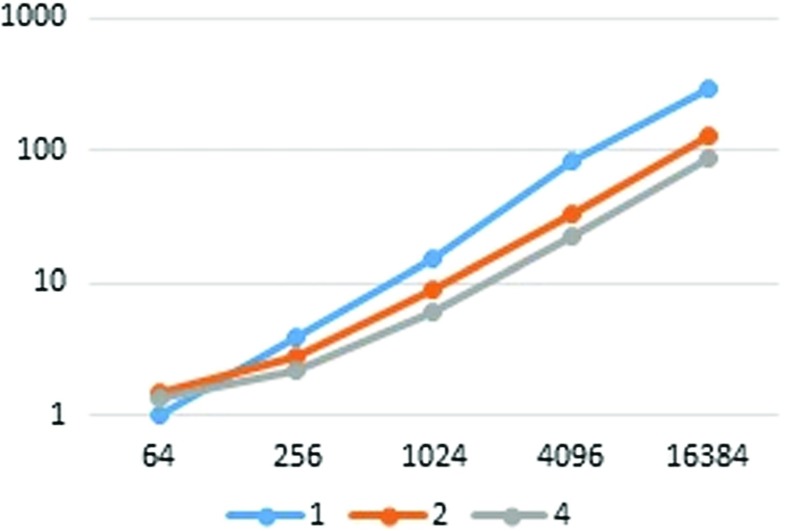
Parallel mode performance (80-bit security)

The performance measurements in parallel mode are presented in Table 7 (Fig. 6 ). We fixed the security parameter at 80-bit security and measured the total runtime, including the computation time and the wait time for communication. Although the total runtimes are not exactly proportional to the number of CPU cores, there is a significant improvement in the multi-core environment. As the time consumption of our protocol is dominated by the encryption and decryption of the Bloom filter array, these processes can easily be implemented in parallel. We believe this property is one of the most important advantages of our protocol.

**Table 7:** Parallel mode performance (80-bit security)

CPU core	Set size				
	2^6^	2^8^	2^10^	2^12^	2^14^
1	1.02	3.89	15.0	82.9	297
2	1.49	2.83	8.72	33.0	131
4	1.33	2.22	6.14	22.6	87.1

All times in the table are in seconds

### Comparison

**Fig. 7:**
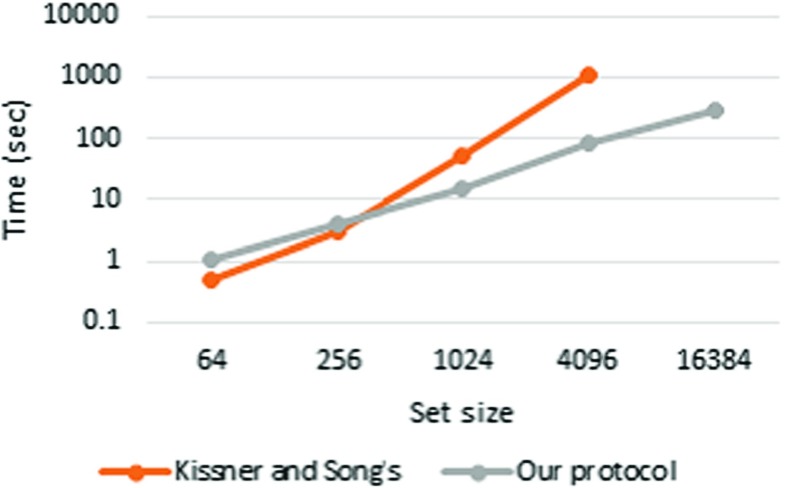
*n*=4

**Fig. 8:**
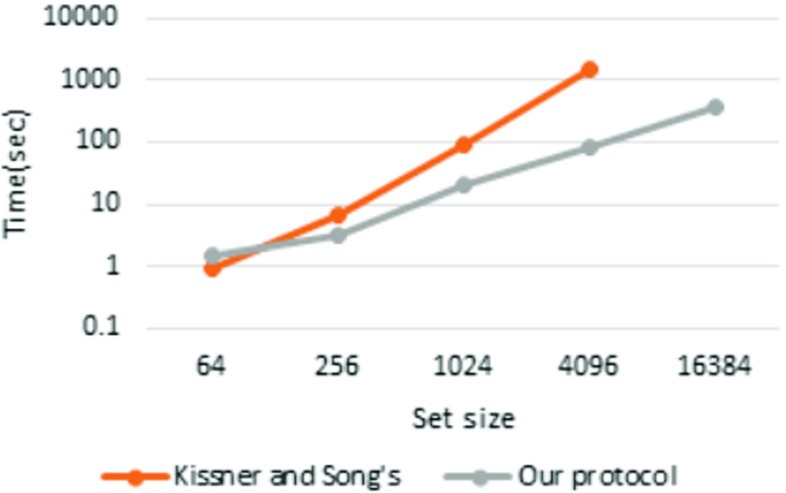
*n*=8

**Fig. 9:**
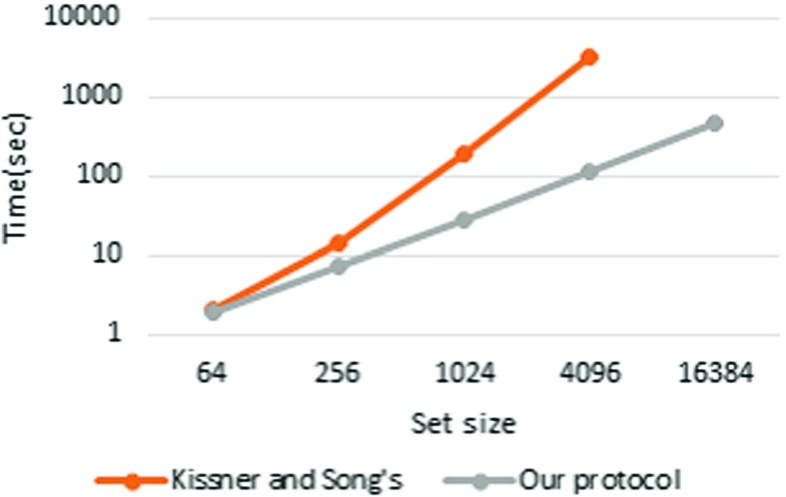
*n*=16

We compared our protocol with Kissner and Song’s MPSI protocol [17]. We implemented Kissner and Song’s MPSI protocol with PARI in C++ for the comparison. All measurements were conducted in pipeline mode. The results are presented in Table 8 (Figs. 7, 8 and 9).

**Table 8:** Performance comparison (80-bit security)

Protocol	Set size				
	2^6^	2^8^	2^10^	2^12^	2^14^
Kissner and Song’s ( *n*=4)	0.50	3.06	50.6	1051	N/A
Our protocol ( *n*=4)	1.02	3.89	15.0	82.9	297
Kissner and Song’s ( *n*=8)	0.92	6.41	92.0	1491	N/A
Our protocol ( *n*=8)	1.50	3.05	19.4	83.2	355
Kissner and Song’s ( *n*=16)	2.10	13.9	190	3246	N/A
Our protocol ( *n*=16)	1.98	7.29	28.7	112	450

All times in the table are in seconds

The results show that our protocol is faster than Kissner and Song’s MSPI protocol when *n*=4 and the set size is greater than 2^8^, when *n*=8 and the set size is greater than 2^6^, and when *n*=16 and the set size is greater than 2^4^. Furthermore, although Kissner and Song’s MSPI protocol crashed with a set size of 2^14^, these results reveal that the time consumption of their protocol is approximately proportional to the square of the set size. As in our protocol, Kissner and Song’s MSPI protocol uses the (*n*,*n*)-threshold scheme, so it does not require a conspiracy assumption. However, their protocol is not scalable with respect to either the set size or number of players.

## Conclusion

This paper has described a practical MPSI in which some of the computations are outsourced to a third-party. As none of the information of *S*_*i*_,|*S*_*i*_|(∀*i*∈[1,*n*]) is revealed to the third-party, this function can be safely outsourced. Our scheme satisfies that the following requirements: any restrictions on the sets are eliminated, meaning that the set size of each player can be flexibly chosen; and the computational burden on each player is independent of the number of players.

Importantly, our scheme can be applied to the efficient integration of medical and related data maintained by different organizations without violating any privacy constraints. We confirmed that the computational complexity is independent of the number of organizations from which data are being integrated.

## Footnotes

1 The computational complexity of *z* for each player can be made independent of the number of players in various ways. For example, set *z*=1. P _1_ computes *z*=*z*⋅*z*_1_ and sends *z* to P _2_, P _2_ computes *z*=*z*⋅*z*_2_ and sends *z* to P _3_, and, finally, P _*n*_ computes *z*=*z*⋅*z*_*n*_ and shares *z* among all players. If we place all players in a binary tree, the communication complexity can be reduced, but each player’s computational complexity is still independent of the number of players.
